# Case Report and Literature Review on Tracheostomal Myiasis: Clinical Presentation, Challenges, and Treatment

**DOI:** 10.1155/crot/1555107

**Published:** 2025-09-27

**Authors:** Yazieed M. Albarrak, Ali M. Alsudays, Mohammed A. Alwabili, Khaled A. Almanea, Fareed R. Alghamdi, Reema A. Aldawish

**Affiliations:** ^1^Department of Otolaryngology Head and Neck Surgery, Prince Sultan Military Medical City, Riyadh, Saudi Arabia; ^2^King Fahad Medical City, Riyadh Second Health Cluster, Riyadh, Saudi Arabia

**Keywords:** maggots, myiasis, otorhinolaryngologists, tracheostomal maggots, tracheostomy

## Abstract

Tracheostomal myiasis, the infestation of a tracheostomy site by fly larvae, is an uncommon and challenging condition, with limited cases reported in the medical literature. This case report aims to describe both the clinical presentation and management of tracheostomal myiasis in a patient with multiple comorbidities. The patient presented with foul-smelling discharge and visible maggots at the tracheostomy site. Management involved manual removal of larvae, surgical debridement, irrigation with iodine solution, and sealing the stoma with paraffin ointment. This case highlights the need for clinician awareness of this rare complication, particularly in patients with compromised health, and highlights the importance of preventive care and early intervention. As a single case report, it reflects the experience within a broader context of managing tracheostomy-related complications.

## 1. Introduction

Tracheostomal myiasis is a rare parasitic infestation of the tracheostomy site caused by fly larvae. It typically affects individuals with compromised hygiene or prolonged tracheostomy, especially those from low socioeconomic backgrounds [1]. The condition is often seen in patients with neurological impairment, immunosuppression, or those living in unsanitary conditions [2].

In neurologically impaired and immunocompromised patients, tracheostomal myiasis occurs due to a convergence of factors that favor larval infestation. Diminished self-care often leads to inadequate tracheostomy hygiene, resulting in the accumulation of secretions, necrotic tissue, and exudate that provide an ideal substrate for fly egg deposition [1, 2]. Inadequate wound care may further promote local tissue maceration or necrosis, enhancing the nutrient-rich environment that attracts flies, while immunocompromised states, whether from chronic illnesses or immunosuppressive therapies, impair local immune responses and hinder effective wound healing [3, 4]. Environmental exposure, particularly in low socioeconomic settings where high fly populations prevail, combined with prolonged tracheostomy exposure and irregular maintenance of the tracheostomy tube, further exacerbates the risk by increasing opportunities for repeated fly contact with the vulnerable stoma [5, 6]. Collectively, these mechanisms underscore the heightened susceptibility of this patient population to tracheostomal myiasis, emphasizing the critical need for meticulous tracheostomy care and caregiver education [7, 8].

Myiasis can be classified as either obligate or facultative. In obligate myiasis, the larvae require living tissue to complete their life cycle, while facultative myiasis occurs when the larvae, usually associated with decomposing organic matter, infest living hosts [3].

The clinical presentation typically includes foul-smelling discharge, visible maggots at the tracheostomy site, and signs of local inflammation, including erythema and swelling [4]. If not managed promptly, complications such as respiratory obstruction, secondary bacterial infections, and even aspiration pneumonia may occur due to larval migration into the airway [5].

The diagnosis is primarily clinical, often made upon visual inspection of the wound. In some cases, imaging techniques such as computed tomography (CT) may aid in assessing the extent of soft tissue involvement, although they may not always reveal the larvae themselves [6].

Management involves mechanical removal of larvae, irrigation with antiseptic solutions, and the application of occlusive agents like paraffin to suffocate residual maggots [7]. Oral antiparasitic agents such as ivermectin have also been employed effectively in some cases to eliminate deep-seated larvae [8]. Preventive strategies include maintaining strict tracheostomy hygiene, regular cleaning, and using protective measures to prevent fly exposure [2].

This case report highlights an instance of tracheostomal myiasis in an elderly patient, emphasizing the clinical presentation, management challenges, and preventive measures to mitigate the risk of such infections.

## 2. Case Report

A 70-year-old woman with a medical history of type 2 diabetes mellitus, hypertension, dyslipidemia, and hypothyroidism was admitted as a case of stroke to the intensive care unit with a low Glasgow Coma Scale (GCS) score. Due to anticipated prolonged intubation, a percutaneous tracheostomy was performed using a size seven cuffed tracheostomy tube. Following clinical stabilization, the patient was discharged with home care support, including caregiver training for tracheostomy maintenance.

**Figure 1:**
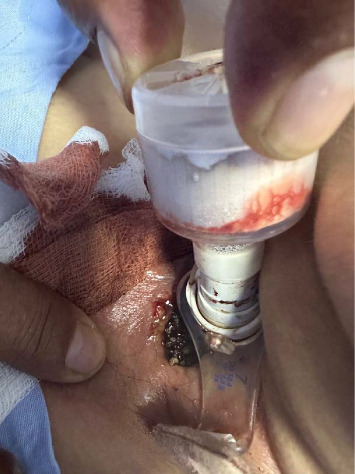
Photograph showing maggots in tracheostomy stoma.

**Figure 2:**
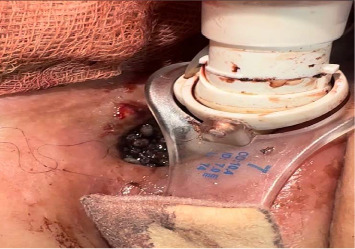
Photograph showing multiple maggots infesting the tracheostomy stoma.

**Figure 3:**
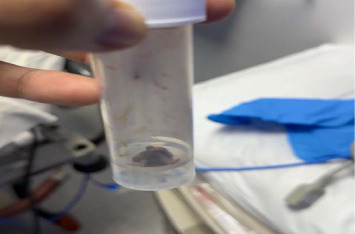
Photograph showing a few maggots in the container after removal sent to the microbiology laboratory.

Seven months later, the patient's caregiver noticed a foul-smelling, red-brown discharge from the tracheal stoma with visible maggots (Figures 1, 2, and 3). The patient was brought to the emergency department. On examination, erythema and discharge were noted around the stoma. The patient was afebrile, and no systemic signs of infection were observed. The family reported adherence to tracheostomy care as instructed but reported low socioeconomic conditions, a recognized risk factor for tracheostomal myiasis.

Upon admission to the otolaryngology department, 34 maggots were extracted manually and sent to the microbiology lab for analysis. The stoma was irrigated with a 1% iodine solution diluted in normal saline, and the site was sealed with paraffin ointment. A contrast-enhanced CT of the neck revealed a small pocket around the stoma without evidence of deep tissue involvement [3]. A flexible fiberoptic bronchoscopy ruled out the presence of maggots within the lower airways.

On the third day of hospitalization, surgical debridement was performed under general anesthesia. The stoma revealed healthy granulation tissue without necrosis. Only four maggots were identified during extensive exploration. The site was irrigated with gentamicin diluted in normal saline. After the procedure, the patient was managed conservatively with continued stoma care and regular antiseptic application.

Two weeks postdischarge, the patient was evaluated in the outpatient clinic. The tracheostomy site remained clean, with no evidence of discharge or recurrent infestation.

## 3. Discussion

Tracheostomal myiasis is a rare but distressing complication of long-term tracheostomy, primarily affecting patients with poor hygiene, impaired self-care, or low socioeconomic status [1, 4, 5]. Flies that lead to myiasis are of two types, either obligate or facultative parasites; obligate as they can only develop in live hosts and facultative being mainly able to survive in cadavers but can sporadically infect live tissue [3]. It has been suggested that percutaneous tracheostomy is a risk factor for developing tracheostomy myiasis [2, 3]. In addition, poor care of the tracheostomy tube and stoma, living in a rural region or having low socioeconomic status, patients with neurological or psychiatric disorders, immunocompromised individuals, wounds that have offensive odor or discharge, exposed wounds, and being in a vegetative state are other risk factors. Early diagnosis is crucial to prevent further tissue damage or complications [4, 5]. In our case, despite caregiver-reported adherence to tracheostomy care, the presence of maggots and discharge highlights the potential impact of socioeconomic factors, a finding consistent with previously reported cases [4, 5]. Previous literature indicates that most cases of tracheostomal myiasis occur in patients with neurological impairment, poor general health, and inadequate personal hygiene [2, 6]. In a study by Bettadahalli et al., tracheostomal myiasis was reported in a child with neurological deficits and poor hygiene practices [3]. Similarly, Failoc-Rojas and Silva-Díaz reported that individuals with compromised consciousness and long-term tracheostomies are at increased risk [4]. Our case aligns with these findings, as the patient's medical history included diabetes, hypertension, dyslipidemia, and stroke, contributing to prolonged immobility and dependence on caregiver support. However, unlike other reports where extensive larval migration occurred [6], in our case, the infestation remained localized, with no evidence of deep tissue invasion or airway involvement, as confirmed by CT imaging and flexible bronchoscopy. However, it is important to note that CT imaging may have limitations in detecting early-stage infestations, as small larvae and superficial involvement can be easily missed. The clinical presentation in our patient included foul-smelling discharge and visible maggots without systemic infection, which mirrors previous reports indicating that localized symptoms are more common than systemic manifestations [4]. The absence of fever or leukocytosis in our patient is consistent with the findings of Kumar et al., who noted that most cases do not present with systemic illness despite the local infestation [6]. The cornerstone of management is the removal of the maggots. With caution, applying insecticides such as chloroform, turpentine oil, and ether at the stoma site while washing and disinfecting the surrounding tissue can also aid in killing larvae and help in the migration of the maggots out of the tracheostomy wound [5, 7]. Management in our case involved manual extraction of 34 larvae, irrigation with diluted iodine, and surgical debridement under general anesthesia. Although oral ivermectin is recommended in some cases due to its antiparasitic properties, it was not administered to our patient as the infestation was localized and there were no signs of systemic involvement. It has been reported that the use of oral ivermectin, a broad-spectrum antiparasitic medication, can help in killing larvae. Moreover, keeping the patient on broad-spectrum antibiotics would help prevent secondary infections. When the patient presents with such a picture, it is advisable to admit the patient for better and proper care and to avoid further complications, such as aspiration of the maggots to the airway that might lead to airway obstruction or aspiration pneumonia [5, 6]. Myiasis infection can also lead to secondary infections such as *Escherichia coli*, *Serratia marcescens*, and *Enterococcus faecalis* [2]. Furthermore, migration or embolization of maggots through blood vessels is another serious complication. Without complications, the overall prognosis is considered to be good [4]. Unlike other reports where recurrence was noted due to inadequate hygiene posttreatment [2], our patient remained infestation free during follow-up, emphasizing the importance of continued education and strict tracheostomy care practices. Overall, our case highlights both similarities and distinctions compared with existing literature. While the clinical presentation and management were consistent with previously reported cases, the absence of systemic involvement and successful prevention of recurrence underscore the importance of comprehensive tracheostomy care and early intervention. Educating caregivers, particularly in socioeconomically disadvantaged settings, remains crucial to minimizing the risk of tracheostomal myiasis. Public health strategies should focus on increasing awareness among caregivers and healthcare providers regarding the prevention and early detection of myiasis, especially in patients with long-term tracheostomies. Addressing socioeconomic factors and providing targeted home care education are vital to reducing the incidence of such infestations.

## 4. Conclusion

Although rare, tracheostomal myiasis can significantly impact patient outcomes if not identified and managed promptly. Clinicians should carefully monitor tracheostomy sites for early signs such as foul-smelling discharge, erythema, or the presence of maggots, particularly in patients with neurological impairments, prolonged immobility, or low socioeconomic status. Educating caregivers on maintaining hygiene, regular tracheostomy care, and recognizing early symptoms is essential to reduce the risk of infestation. As this is a single case report, further documentation through multicenter studies is necessary to understand better the risk factors, optimal management strategies, and preventive measures for tracheostomal myiasis.
